# Silver Nanoparticle-Based Nanocomposites for Combating Infectious Pathogens: Recent Advances and Future Prospects

**DOI:** 10.3390/nano11030581

**Published:** 2021-02-26

**Authors:** Md A. Wahab, Luming Li, Hongmei Li, Ahmed Abdala

**Affiliations:** 1 Institute for Advanced Study, Chengdu University, Chengdu 610106, China; liluming@cdu.edu.cn (L.L.); lihongmeihappy@126.com (H.L.); 2 College of Food and Biological Engineering, Chengdu University, Chengdu 610106, China; 3 Chemical Engineering Program, Texas A&M University at Qatar, Doha POB 23874, Qatar

**Keywords:** antibacterial, infectious diseases, nanoparticles, silver, biopolymers, chitosan, disinfectant, water and air treatment

## Abstract

Silver nanoparticles (Ag NPs) and their nanocomposites with polymers are potent agents for antibacterial and disinfectant applications. The structural parameters of Ag-NPs, such as size, shape, and surface area, are very critical for developing appropriate formulations for the targeted applications. The impact of these factors on the performance of Ag NPs is analyzed. Ag NPs with a broad spectrum of antibacterial activities have already found applications in wound and burn dressing, food preservation, agricultural ponds, treatment for infected areas, coatings, water treatment, and other biomedical applications. Ag NPs are quite useful against antibiotic-resistant bacteria, but their level of toxicity needs careful investigation as their toxicity could be very harmful to human health and the environment. This review discusses the challenges and prospects of various Ag NPs and their composites. The review will enrich the knowledge about the efficiency and mechanism of various Ag nanoparticle-based antibacterial agents.

## 1. Introduction

Although significant progress has been achieved in public health, there is a potential disease threat caused by infectious pathogens [1,2,3,4,5,6]. Nowadays, various antibacterial agents, including antibiotics, have been widely employed in treating diseases caused by these infectious pathogens. However, the excessive use of such agents has developed drug resistance, resulting in low treatment efficacy and significant economic loss. The development of antibiotic-resistant bacteria has received much attention globally [1,2,3,4,5,6,7]. Carbapenem-resistant Enterobacteriaceae (CRE) bacteria could kill up to 50% of the patients who get bloodstream infections. CRE is dangerous bacteria, and even the most potent antibiotics have failed against it, leaving infected patients helplessly with “like untreatable infections” [4]. Nowadays, hospital infections have increased by two-thirds because of *Escherichia coli* (*E. coli*) and *Klebsiella* bacteria [5].

Most importantly, these infectious pathogens are rapidly spreading from patient-to-patient, surface-to-surface, surrounding environments, and among health care workers and patients [4,5,6]. Currently, microbial-based infections are one of the critical issues because of these infections; a large number of people are helplessly dying in different countries. The growing resistance of microbes to antibiotics and antifungal treatments suggested imperatively to search for alternative antibacterial materials that are resistant to colonization. The detailed analysis of the interaction of bacteria in films with the employed antibacterial agents was also reported by Costerton et al. [8]. There have been many studies to overcome bacterial and other infections [8,9,10,11,12,13,14,15,16,17,18,19,20,21,22,23,24,25,26]. In this context, there is a pressing need to develop novel, more efficient, and cost-effective antibacterial agents.

Recently, the modifications of various metallic nanoparticles (NPs) from bulky/micro-size to nano-size scale have shown excellent ability to control the properties and have resulted in tremendous progress, which has created huge interests in a wide range of applications [27,28,29,30,31,32,33,34,35,36,37,38,39,40]. The correlation between the NPs composition, size, and shape and their bioactivity and the biological response is being investigated [27,28,29,30,31,32,33,34,35,36,37,38,39,40,41,42,43,44,45]. In the past several years, inorganic NPs have received much attention as potential antibacterial materials in the fields of biomedical and environment owing to their controllable size and shape along with excellent reactivity and unique physical, chemical, and biological properties [1,2,3,7,8,9,10]. Inorganic NPs with antibacterial activities, including iron [1]. zirconium [13], gold [12,14], titanium [9], silica [7], copper [15], zinc [14], and silver [7,8,9,10,11,12,13] are reported to minimize infections caused by infectious pathogens. Among these inorganic NPs, Ag NPs have been widely researched because of their broad spectrum of antimicrobial activity. The bactericidal effect of Ag NPs could be tuned by their size, shape, distribution, aggregation, chemistry, surface coatings, and support matrix [1,2,3,4,5,29].

Very recently, Pallavicini et al. [29] have demonstrated photothermal antibacterial properties of Ag NPs as surface coatings. In their paper, the intrinsic and switchable photothermal activities of these surfaces and their synergistic effects were thoroughly discussed [29]. Ag NPs are frequently present in the form of large aggregates in the range of 70 nm to 400 nm with a low surface area. Such large aggregates of NPs reduce their effectiveness to inhibit the growth of infectious pathogens [3,4,5,6,7,8,9,10]. Ag NPs with a size of 10–15 nm embedded in a support matrix is more effective because their small size allows them to penetrate the bacterial cells easily and ultimately damage the cells by creating pores on their walls [4,5,6,7,8,9,10,11,12,13,14,15,16,17,18,19]. The larger surface area of NPs provides higher antibacterial efficiency and can inhibit biofilm formation. Additionally, Ag NPs have demonstrated good antimicrobial activity because of the unique physicochemical characteristics of Ag, as well as the large specific surface area that provides the microbes with more exposed surface, resulting in higher antimicrobial activity [1,2,3,11,12,13,14,15].

Silver nanoparticles (Ag NPs) play a vital role in several applications, including air and water disinfection, biomedical fields, textile, and health care products, wound dressing/healing, and food packaging. Moreover, Ag NPs have already shown profound bactericide effects against *Escherichia coli*, *Pseudomonas aeruginosa*, *Bacillus cereus*, *Staphylococcus aureus*, and *Salmonella choleraesuis* bacteria, which were associated with the higher cell toxicity [4]. Ag NPs are very effective in terms of efficiency due to their stronger ability to bind to sulfur and phosphorous parts of the bacteria to kill the cells [5]. Moreover, the potential risks are associated with the presence of NPs in the living organisms if any retention occurred. For example, antimicrobial NPs could act intra- or extracellularly depending on their nature and size. The NPs surface chemistry and their tendency to aggregate dictate their risk factor. Therefore, toxicity studies should be systematically investigated before NPs are introduced into the health care sectors, such as antimicrobial treatments or barriers or coatings or surface use. This article reviews the recent progress on the applications of Ag NPs as antibacterial agents to combat infectious diseases. Moreover, possible inhibition mechanisms and side effects of Ag NPs are analyzed.

## 2. Application of Ag NPs

### 2.1. Antibacterial Activity of Ag-NP-Based Biocomposites

**Figure 1:**
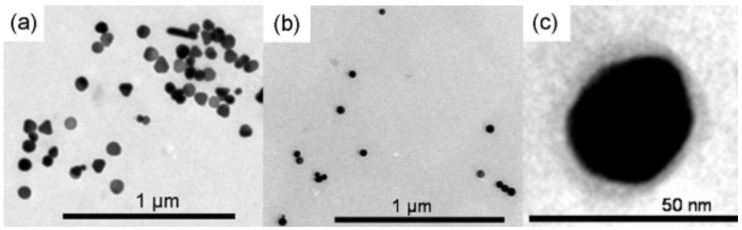
TEM images of the as-prepared silver-chitosan nanoparticles (Ag-CS NPs) with 16.5 mM trisodium citrate (TSC) concentration (**a**) at 35 ± 2 °C, (**b**) at 0 °C, and (**c**) higher magnification of (**b**) showing an Ag NP coated by CS (adapted with permission from reference [10]).

Due to the vital antibacterial activities of Ag-NPs against a wide range of microorganisms, the development of Ag NP biocomposites has received significant attention [7,8,9,10,11,12,13,14,15,16,17,18,19,20,21,41,42,43,44,45,46,47,48,49,50,51]. For example, chitosan (CS) biopolymer, which is biocompatible and non-toxic, has had its antibacterial activity explored against Gram-negative and Gram-positive bacteria [7,8,9,10,11,12,13,14,15,16,17,18,19,20,21]. Monica et al. reported a new method to incorporate Ag NPs into CS biopolymer [10]. Figure 1 shows the TEM images of Ag NPs with 43–55 nm in sizes. Figure 1c confirms that Ag NPs are coated with the CS biopolymer layer.

**Figure 2:**
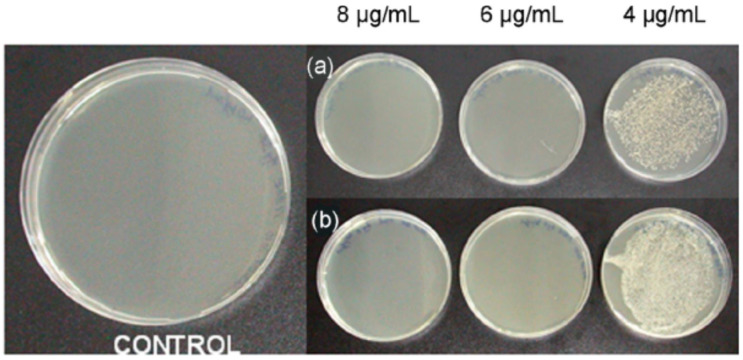
Testing the bactericidal activity of CS-Ag NPs prepared at 0 °C against (**a**) *Staphylococcus aureus* UCLA 8076 and (**b**) *S. aureus* 1190R. The left panel shows the control sample (adapted with permission from reference [10]).

Stable CS-coated Ag NPs are reported as highly effective against Gram-positive *S. aureus,* as shown in Figure 2 [10]. The results of the study suggested that the use of a combination of CS and Ag NP-based composite can impact more significantly against *S. aureus* than either Ag NPs or CS polymer alone due to the synergistic action of the two components [10]. Moreover, coating the NPs with a biopolymer leads to steric stabilization and decreases the potential aggregation tendency, suggesting an enhanced effective concentration of the NPs that are capable of interacting with the cellular surface. The positive charge by the CS promotes binding capacities to negative charges present at the cell surface [10].

Another study has prepared a biocomposite using CS biopolymer and Ag NPs via an environmentally friendly in situ chemical reduction process, and spherical-shaped Ag NPs with 3 to 11 nm were synthesized. These biocomposites of CS and Ag NPs have very effective antibacterial activity against *S. aureus* and *E. coli* [16].

**Figure 3:**
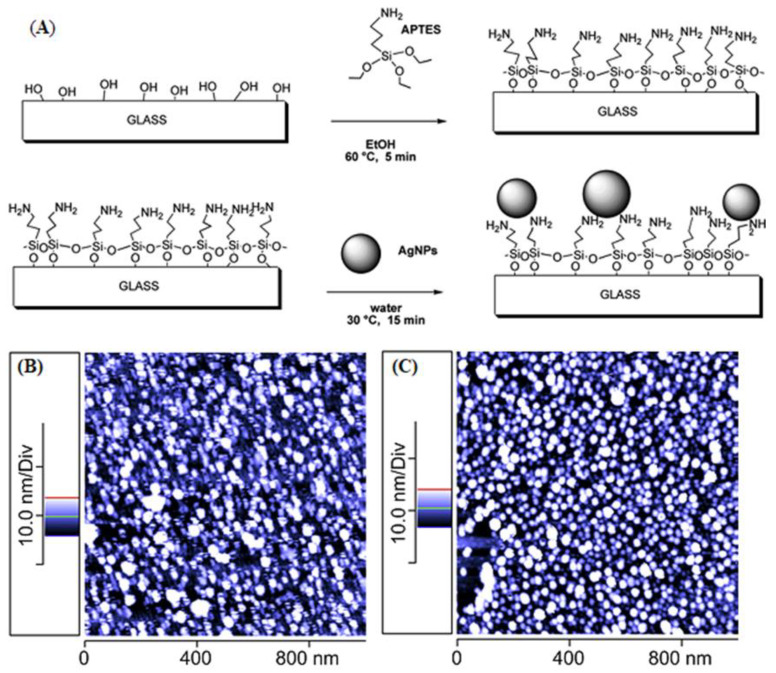
(**A**) Schematic diagram of making amine-functionalized glass substrate; (**B**) atomic force microscopy images (AFM) taken from a freshly prepared glass slide grafted with Ag NPs after silanization with aminopropyl silane (APTES) and (**C**) AFM image of the same glass slide kept in bidistilled water for 20 days (adapted with permission from reference [14]).

Taglietti et al. [14] modified glass substrate with aminopropyl silane (APTES), and then deposited Ag NPs on that modified glass surface through coupling with the amine functional groups as schematically depicted in Figure 3A. TEM analysis revealed the formation of Ag NPs with less than 10 nm size, which was also confirmed by atomic force microscopy (AFM) (Figure 3B). Moreover, the Ag NPs produced via this new method were able to inhibit the biofilm growth caused by *Staphylococcus epidermidis* RP62A [14].

A composite of biopolymers containing 1 wt.% Ag NPs was reported to form aggregated particles, and their dispersion was dependent on the solvent, the Ag NP concentration, and the used biopolymer [8,9,10,11,12,13,14,15]. For example, Ag NPs with 2.7 ± 1.5 nm in biodegradable polycaprolactone fibers have entirely inhibited the growth of *E. coli*, *Listeria monocytogenes*, and *Salmonella typhymurium* [15]. Pallavicini et al. [32] have described the antibacterial and antibiofilm formation activities of Ag NPs using AgF as the starting source for Ag NPs and pectin as a reductant and protecting agent as well. The produced Ag NPs from the AgF exhibited an enhanced antibacterial activity against *E. coli* PHL628 and *Staphylococcus epidermidis* RP62A due to the synergy between p-Ag NPs and F^−^ anion [32].

### 2.2. Wound Dressing and Orthopedic Coating

The fabrication of a Ag NP-based biocomposite with significant antibacterial activity depends on the nature of the materials employed in the preparation process and the synthesis conditions that will impact the anticipated properties of the end products [7,8,9,10,11,12,13,14,15,51,52,53]. The functional properties, such as pore size, swelling index, non-toxicity or biocompatibility, biodegradation, and mechanical strength of the Ag NP-based biocomposites, play a key role in designing materials for wound-dressing applications. Wounds such as burns, extensive burns may lead to extensive damage to the skin. Such burns might have the potential risk of loss of body fluid and acute inflammation [7,8,9,10,11,12,13,14,15,51,52,53]. When wounds are infected, the overall healing process will be more complicated and impeded. Some of the bacterial infections require external medicines [7,8,9,10,11,12,13,14,15,51,52,53]. Moreover, a dressing pad impregnated with a broad-spectrum antibacterial agent that has a high bacterial-killing rate and curing is one of the sound options. In this context, Ag NPs can be an excellent broad-spectrum antibacterial material that can be easily incorporated within a biopolymer via preparing biopolymer-Ag nanocomposite because of their biocompatibility, good flexibility, tear-resistance, and antimicrobial activity [8,9,10,11,12,13,14,15,16,17,18,19,20,21,51].

**Figure 4:**
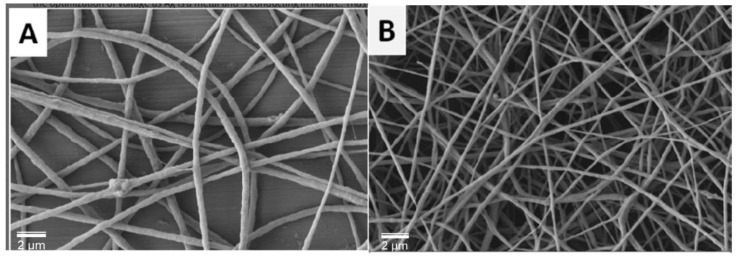
SEM images of (**A**) poly(ethylene oxide) (PEO)–PCL-blended nanofiber and (**B**) PEO-PCL/Ag NPs composite nanofibers (adapted with permission from reference [17]).

Gopinath et al. [17] have developed fibrous biocomposites for wound-dressing applications based on incorporation of different concentration of Ag NPs. As shown in Figure 4, the composites were highly fibrous nanostructured fibers, which were prepared from poly(ethylene oxide) (PEO)–polycaprolactone biopolymer. The diameter of these PEO–PCL nanofibers containing Ag NPs (15–20 nm) ranged from 70–150 nm.

**Figure 5:**
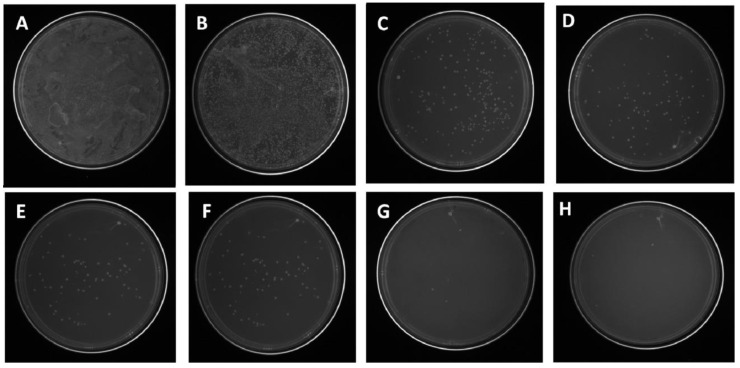
Effects of nanofiber concentrations on the viability of antibiotic-resistant GFP *Escherichia coli*: (**A**) PEO–PCL nanofiber and (**B**–**H**) 1, 2, 4, 6, 7, 8, and 10 mg of composite nanofiber (adapted with permission from reference [17]).

The effects of the nanofiber concentration on the viability of antibiotic-resistant GFP *E. coli* were investigated, and the results are shown in Figure 5 [17]. The images for various concentrations in Figure 5 suggest the deformation of the bacterial cell morphology, confirming the penetration of Ag NPs into the cells of the GFP *E. coli,* leading to the complete degradation of the bacterial cell wall within 12 h [17]. It is also reported that the binding of NPs to the bacterial proteins and the generation of reactive oxygen species (ROS) would preclude the cell death. The developed composite nanofibers have shown good roughness, wettability, and antibacterial activity against recombinant green fluorescent proteins expressing antibiotic-resistant *E. coli*, suggesting that composite materials could be a potential candidate for wound dressing [17]. These results were supported by other reported works [51,52,53]. Very recently, pectin with a low content of ester was used to prepare pectin-stabilized spherical Ag NPs from AgNO_3_ as an Ag^+^ source [33] and test them against *E. coli* PHL628 (*E. coli* PHL628, Gram-negative) and *Staphylococcus epidermidis* RP62A (*S. epidermidis* RP62A, Gram-positive). Moreover, these Ag NPs are tested on fibroblasts and the results suggested their potential application for wound healing [33].

**Figure 6:**
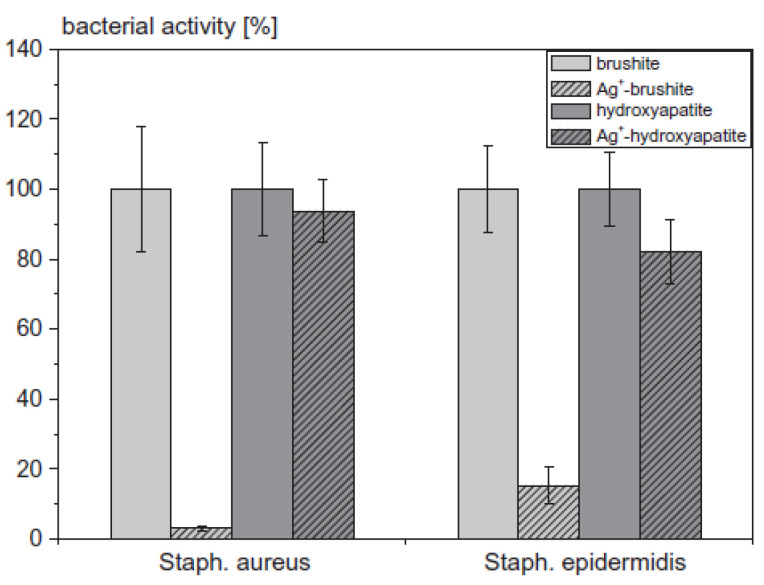
The bacterial activity of *S. aureus* and *Staphylococcus epidermidis* on cement surfaces (n = 4) determined by the WST-1-test in LB medium (adapted with permission from reference [58]).

Despite the advances made in clinical approaches, implanting a metal-based orthopedic device with long-term survivability remains a challenge for orthopedic surgeons due to the risk of microbial infection, which becomes more complicated in cases such as open-fractured bones and when any bacterial biofilm forms on the implant site [18,53,54,55,56,57,58,59,60,61,62]. It is also safely suggested that infected tissue can infect the next attached tissue that could be part of another organ of the body, which is known as “side effects due to such infection” [55,56,57]. Gbureck et al. [58] have evaluated a ceramic bone cement loaded with Ag NPs (Ag^+^) against Gram-positive *S. aureus* and *S. epidermidis.* The results, Figure 6, suggest Ag^+^-hydroxyapatite has better antimicrobial activity associated with the released Ag^+^ from the cement matrices in both phosphate-buffered saline (PBS) and Luria broth (LB) medium.

Sometimes, due to the impaired blood circulation at the injury site and low local concentration of drugs, the administrative drug may not be as effective as required, and an infected site will take a long time to be cured. In this case, there would be a chance of getting infected again leading to further complications. To overcome such problems, meanwhile, various kinds of Ag NP-based composite coatings have been reported [53,54,55,56,57,58,59,60,61,62]. As discussed before, one of the metal nanoparticles, particularly Ag NPs with a broad spectrum of antibacterial activity, could be very effective against Gram-positive *S. aureus, S. epidermidis,* and Gram-negative *E. coli*, *Pseudomonas aeruginosa* bacteria [1,2,3,4,5,6,7,63,64,65,66,67,68,69,70,71].

**Figure 7:**
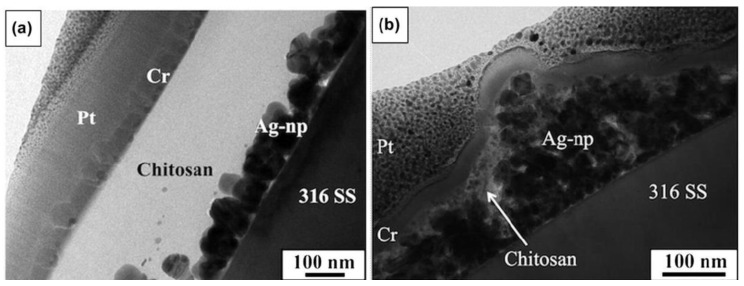
TEM cross-sections of (**a**) CS/Ag and (**b**) CS/Bioglass (BG)/Ag films (adapted with permission from reference [18]).

Boccaccini et al. [18] developed a composite orthopedic coating using CS and Ag NPs via a single-step electrophoretic deposition technique. The TEM images in Figure 7a demonstrates that CS/Ag NPs composite coating with 50–80 nm thickness is coated with a CS layer. In contrast, a thicker layer of finer-size Ag NPs (50 nm) is infiltrated with the CS layer, as shown in Figure 7b [18].

**Figure 8:**
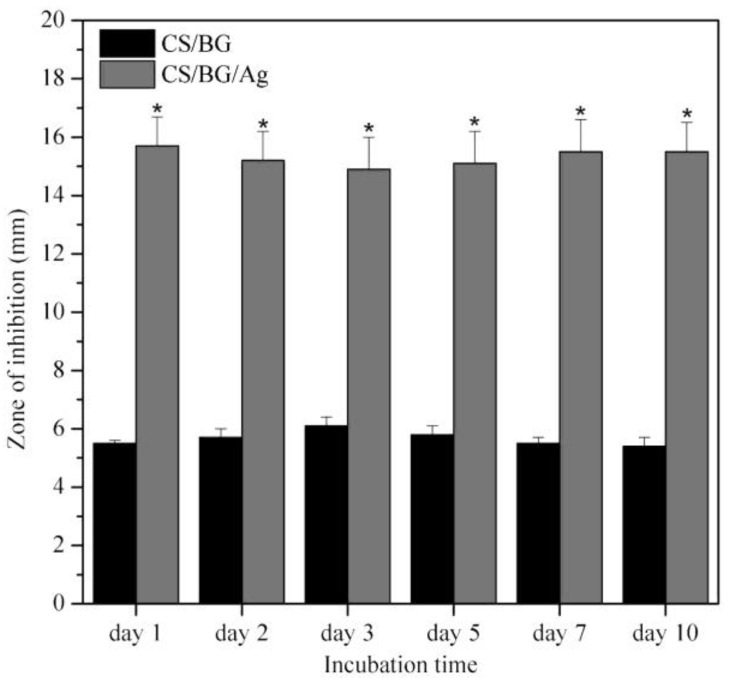
Evolution of the size of the inhibition zone after immersion in phosphate-buffered saline (PBS) of CS/BG (Chitosan/Bioglass) and CS/BG/Ag (Chitosan/Bioglass/silver nanoparticles): The data represent mean ± SD of three individual experiments (* *p* < 0.05 CS/BG/Ag vs. CS/BG. (adapted with permission from reference [18]).

**Figure 9:**
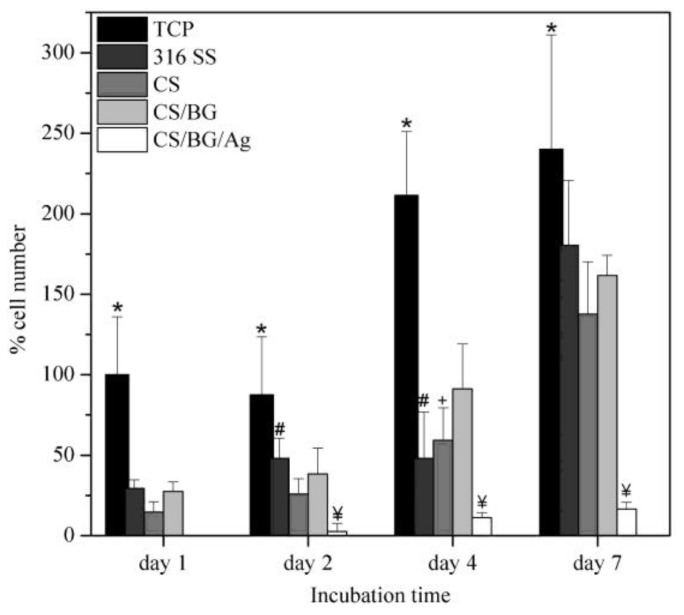
Effect of coating SS-316 (stainless steel-316) with CS (chitosan), CS/BG (Chitosan/Bioglass), and CS/BG/Ag (Chitosan/Bioglass/silver nanoparticles) on MG-63 cell viability measured over seven days by alamarBlue^(R)^ assay. Data represent mean ± SD of two individual experiments each performed in quadruplicate. (*p* < 0.05 at the same time period: * is for TCP vs. all other coatings; ¥ is for CS/BG/Ag vs. all other coatings; # is for marked bar vs. CS; + is for marked bar vs. CS/BG) (adapted with permission from reference [18]).

The CS/bioglass (BG) and CS/BG/Ag nanoparticles are tested for antibacterial studies with SS-316 as standard metallic orthopedic substrate. While the PBS control, SS-316, and CS did not develop any inhibition zones, both CS/BG and CS/BG/Ag NPs exhibited antimicrobial activities as revealed by the size of the inhibition zone shown in Figure 8 [18]. It is also worth noting that the CS/BG/Ag NPs were more effective than CS/BG, as indicated by the size of the inhibition zone, which is three times more than CS/BG. The cellular metabolic activity in Figure 9 also shows that CS, CS/BG, and the controls (TCP and SS-316) supported the propagation of the MG-63 cells over the seven days. At each time point, it is noted that all coatings have shown significantly smaller cell numbers (*p* < 0.05) than those of the positive control sample.

As shown in Figure 9, there is no significant difference among the three samples (316 SS, CS, and CS/BG). The significantly lower percentage of cells on CS/BG/Ag coating in comparison with all other coatings is notable, indicating the increased levels of active Ag in the composite structure that lead to cellular toxicity. The study also suggested the sustained release of Ag^+^ ions from the composite coating that could facilitate bactericidal activity in vitro as supported by the slow release of Ag ions that have killed *S. aureus* [18].

### 2.3. Disinfectant

Despite the advancements in many fields in the last several decades, a sufficient supply of freshwater is still among the top vital goals to be accomplished by 2030 [1,2,3,72]. Meanwhile, various approaches are employed to provide freshwater sources. For example, filtration has already been widely used, which can remove only suspended or microparticles from water. The filtration depends on the size of the filler and employed materials. On the other hand, another technique is the advanced oxidation treatment, which is usually employed to degrade organic components in contaminated water. This method does not remove all organic components from contaminated water. Another method, such as reverse osmosis, was used to purify water, but it was a costly process that limited its widespread application.

On the other hand, water filters with UV-exposure are used for the disinfection of water from microorganisms. However, multifunctional systems are required to decompose moieties and function against microbes present in water simultaneously. Such systems would become more economical and feasible. Among the various NPs, composites of Ag NPs with their broad spectrum of antimicrobial activities provide innovative formulations with multitasking ability against various microorganisms. In water treatment, Ag NPs can treat water by both degrading the organic substances and killing the different biological species in one step without affecting the water physicochemical properties [73,74].

Because of its multi-domain uses, Ag NP-based disinfectants have received much attention for water and air. The US Environmental Protection Agency (EPA) and WHO have recommended that the Ag concentration of < 0.1 mg/L in drinking water is safe [75]. The disinfection ability of Ag depends on the various experimental conditions, including concentration, pH, size, exposure time, temperature, and other components, including calcium, sulfide, and chloride present in water. Among these other components, chloride ions have less impact on the Ag bactericidal activity compared to calcium and sulfide ions [76]. Ag can form nanocrystalline that can provide better efficacy in water disinfection than regular filters [77]. Filters impregnated with Ag NPs should show simultaneous functionality such as disinfectant and physical filtration. Ag salt and its NPs are effective for disinfection against *C. parvum* microorganisms and its subsequent removal from water by filtration, indicating the dual role of Ag NPs [78].

**Figure 10:**
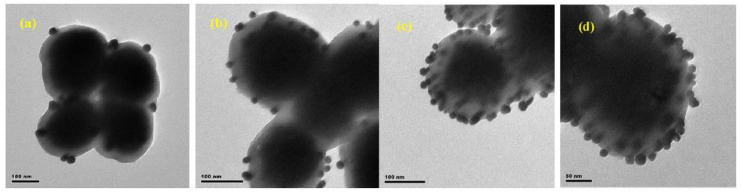
TEM images of (**a**) Fe_3_O_4_@PTA@Ag-1, (**b**) Fe_3_O_4_@PTA@Ag-2, (**c**) Fe_3_O_4_@PTA@Ag-3, and (**d**) Fe_3_O_4_@PTA@Ag-4 (adapted with permission from reference [79].

Wang et al. [79] have produced magnetic microspheres loaded with Ag NPs. These microspheres have a good disinfectant capability with high antibacterial activity for water disinfection. In that study, the layer from tannic acid-metal polymer was employed to prepared various Fe_3_O_4_@PTA@Ag nanocomposites. TEM analysis of the magnetic microspheres are shown in Figure 10. The TEM analysis reveals the presence of 19 nm Ag NPs inlaid on the surface or deeply embedded into the interior of the magnetic microspheres. Despite maintaining the same shape, the Ag NPs have a size distribution that is dependent on the loading of Ag NPs [79].

**Figure 11:**
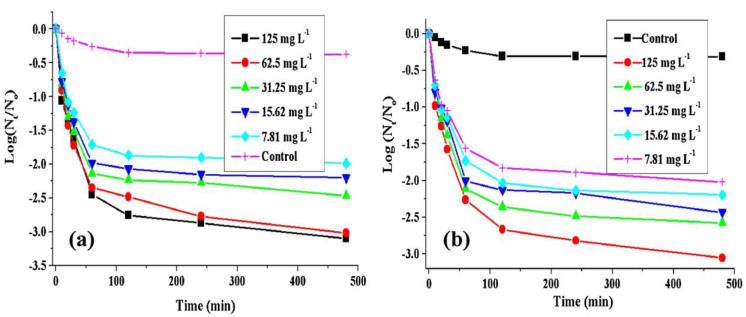
The inactivation curve of Fe_3_O_4_@PTA@Ag-3 toward *E. coli* (**a**) and *S. aureus* (**b**) (adapted with permission from reference [79]).

The effect of Fe_3_O_4_@PTA@Ag-3 concentration on the evolution of the sterilization percentage for *E. coli* and *S. aureus* is shown in Figure 11 [79]. The presence of Fe_3_O_4_@PTA@Ag-3 destroys more living cells. More than 99% of the viable cells was deactivated after 1 h of contact time using 1.25 g L^−1^ of the magnetic disinfectant. In contrast, due to the differences in the cell wall structure and the composition of the Gram-negative (*E. coli*) and the Gram-positive (*S. aureus*) bacteria, Fe_3_O_4_@PTA@Ag-3 exhibited higher inactivation ability against *E. coli* than against *S. aureus*. This higher inactivation ability is attributed to the presence of the thick and rigid peptidoglycan layer in the cell wall of the Gram-positive bacteria that causes some resistance to Ag NPs. Thus, incorporating Ag NPs in materials such as ceramic filters, polyurethane foams, and blotting papers with other metal particles will help design new nano- or micro-based devices for water purification.

### 2.4. Other Antibacterial Applications

**Figure 12:**
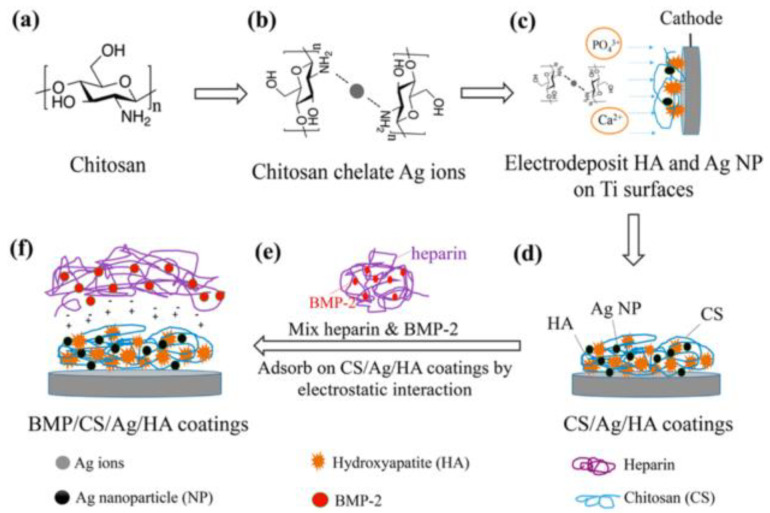
Schematic diagram of the different steps for the electrochemical deposition and immobilization of (bone morphogenetic protein -2 (BMP-2) on hydroxyapatite (HA) coatings (adapted with permission from reference [63]).

The broad-spectrum of antibacterial activity of Ag NPS compared to other common antibiotics will bring benefits to medical applications, including urology, dentistry, general surgery, and orthopedics [20,63,64,65]. Vasile et al. [20] have prepared polyurethane (PU)–extracellular matrix (EM)/silver biocomposites. The presence of metallic Ag NPs in the PU membrane was confirmed by XPS and XRD, while Ag only in the oxidized state was found in the PU-EM membranes. These materials were undergone in vitro antimicrobial tests against a few pathogens such as *E. coli, Salmonella typhymurium*, and *Listeria monocytogenes* and results have revealed the inhibited bacterial growth against these infectious pathogens [20]. A low amount of biocide and the relatively cost-effective process suggest the possible application of urinary catheters [20]. Fang et al. [63] synthesized hydroxyapatite (HA) coatings, as shown in Figure 12, based on Ag NPs and growth factors. These materials were used for the enhancement of osteoinductivity and antibacterial activity [63]. Osteoblast (OB) cultures revealed the biocompatibility and effectiveness of the material against *S. epidermidis* and *E. coli* [63]. A bone marrow stromal cells (BMSCs) culture was also conducted, and the results have shown the BMP/CS/Ag/HA coatings to have better osteoinductivity and enhanced differentiation of BMSCs [63].

CS was used as a stabilizing agent to making homogeneous distribution of Ag NPs on the HA coatings. Notably, CS has played two roles: (i) CS decreased the toxicity of Ag NPs without compromising its antibacterial ability, and (ii) CS has also significantly facilitated the immobilization process of BMP through the electrostatic interaction between biomolecules [63].

A facile and efficient approach for preparing antibacterial HA coatings using Ag NPs was reported, and the approach can be extended to prepare a variety of metallic implant surfaces [64]. The materials coated with Ag NPs were added to water filters for effective removal of *E. coli* [64]. On the other hand, homogenously distributed Ag NPs over chemically reduced graphene were effective for removing the coliform bacteria from sewage water taken from highly polluted regions [65].

## 3. Adverse Effects

Because of their antimicrobial activity that can be tailored by merely altering their size and shape, Ag NPs have applications in several different areas, including food packing materials (10.04%), health and fitness (52.61%), cleaning (10.44%), electronics devices (3.21%), household appliances (6.02%), toys (2.01%), medical appliances (4.02%) and other (11.65%) [72,80,81]. Because of these widespread applications, it is necessary to raise awareness of the adverse effects of Ag NPs on human health and the environment ecosystem. Therefore, research on the impact of Ag NPs on the health and environment, as well as the long-term risk associated with using such NPs, should be investigated thoroughly. Eventually, risk-assessment studies are expected to fulfill the knowledge gap of toxicity.

Ag NPs with different sizes function differently against different organisms. Therefore, Ag NPs will have different degrees of toxicity associated with them depending on their size and concentration [1,2,3,14,72]. For example, higher doses of Ag NPs will release more Ag NPs that could be easily absorbed by the system, such as aquatic species. The excessive use of Ag NPs as a disinfectant could create microbial resistance, which is again against our expectations. Thus, it might also lead to severe health complications [1,2,3,7,24]. Therefore, caution in use of Ag NPs is recommended, and further investigation of the mechanism of their action and toxicity is necessary for avoiding long term consequences.

## 4. Inhibition Mechanisms of Ag NPs against Bacterial Growth

The possible antibacterial mechanisms of Ag NPs will be discussed in light of the available studies, as the type of Ag NPs (size, shape, concentration, surface area, and surface charge) impacts the inhibition growth of bacteria [1,2,3,7,8,9,10,11,12,13,14,15,16,17,18,19,20,21,24,65,66,67,68,69,70,71,72,73,82,83,84,85,86,87]. For example, Ag NPs with the same surface area will predominantly show a shape-dependent interaction with *E. coli*. In contrast, different shapes of Ag NPs display various effective surface areas, but active facets of the particle will play a role in altering the interactions with microorganisms [1,2,3,7,8,9,10,11,12,13,14,15,16,17,18,19,20,21,24,82,83,84,85,86,87].

Raza et al. [84] synthesized two types of Ag NPs via a wet chemical method. The first type was spherical Ag NPs with 15–90 nm, and the second type was triangular Ag NPs with ~150 nm size. All synthesized Ag NPs were tested against Gram-negative bacteria, i.e., *Pseudomonas aeruginosa,* and *Escherichia coli.* The zone of inhibition (ZOI) results indicated the smallest-size, spherical Ag NPs have better antibacterial activity against both bacteria strains than the triangular and larger spherical-shaped Ag NPs. The higher activities of the smaller-size Ag NPs are attributed to their ability to penetrate inside bacterial cells more efficiently than the larger-size, triangular-shaped Ag. The study reported that inside the bacteria, the spherical Ag NPs, being a weak acid, possibly interact and destroy the sulfur- and phosphorus-containing complexes (soft bases) like DNA, and also disrupts the morphology of the membrane, finally leading to the cell death [84]. These results were consistent with previously published results [82,85,87,88,89].

Based on the XRD analysis, spherical Ag NPs had the most intense diffraction peak that is associated with the basal plane of the {111} facets [84]. Therefore, spherical-shaped Ag NPs have the high-atomic-density of the {111} facets along with the large surface to volume ratio. Such facets are reported to be more active sites for enhancing bacterial killing efficiency compared to triangular-shaped Ag NPs [84]. Other studies reported the interaction of different facets with bacteria surface [82,87]. Morones et al. [82] have demonstrated particles with 1–10 nm size (98%) have highly reactive {111} facets. Ag NPs of < 10 nm size have the most remarkable ability to attach to the sulfur-containing membrane and consequently have the maximum permeability through the membrane and eventually the highest ability to cause the death of the bacteria cells [82]. These findings are consistent with the report of Lu et al. [86], who synthesized three sizes of stable Ag NPs (5, 15, and 55 nm). All these Ag NPs were tested against five anaerobic oral pathogenic bacteria, Gram-positive *S. mutans*, *S. sanguis*, *S. mitis,* and Gram-negative *A. actinomycetemcomitans, F. nuceatum,* and aerobic bacteria *E. coli*. The Ag NPs with 5 nm size provided the highest antibacterial activity. Therefore, it is evident that the size and shape of Ag NPs dictate their antimicrobial activity [85,86,87,88,89,90].

On the other hand, the bacterial cell membranes have sulfur that constitutes proteins and sulfur with amino acid functional groups. Ag NPs can easily interact with the inside and outside of the cell membrane, and inactivate the bacteria through the inhibition growth process. Ag NPs also interfere with the way bacteria replicate their genetic structure, preventing further proliferation, possibly as a result of oxidation of DNA by reactive oxygen species (ROS) inside the cells [7,8,9,10,11,12,13,14,15,21,24,73]. Moreover, Ag^+^ ions released from Ag NPs interact with the DNA phosphorus and the sulfur-containing proteins, which could lead to inhibition of the enzyme activities [82].

Recently, the incorporation of Ag NPs of less than 10 nm size was able to puncture the cell wall, creating pores/holes through which the cytoplasmic is released into the medium, leading to the death of the cell without any further interaction with the bacteria intra- and extracellular proteins and nucleic acids [82,83,85,86,87]. Such interactions of the Ag NPs with some cells will initiate apoptosis, which is the form of programmed cell death, indicating injured cells will die eventually. Other studies on the inhibition of bacteria growth found Ag^+^ to inhibit the cell growth by coordinating with and oxidizing the thiol groups of the vital bacterial enzymes [66,67,68,69,70,71,72,73]. Thus, we conclude that the antibacterial mechanism of Ag NPs is a multifaceted process, including interruption of transmembrane electron transfer, disruption of the cell envelope, and oxidation of cell proteins and nucleic acids via the intermediacy of ROS [1,2,3,83].

## 5. Conclusions and Perspective Directions

To reduce the risk of microbial infections, Ag NPs have become one of the alternative agents over the last several years. Ag NPs are effective in treating infectious illnesses, even targeting specific areas, including hard-to-reach sites in which pathogens can harbor. Treating these pathogens with Ag NPs allows the Ag NP to penetrate the cell wall (creating a pore on the cell wall) and non-specifically interfering with essential cellular biochemistry. Ag NP-biopolymer composites were also used against infectious pathogens (such as *E. coli*, *S. aureus,* etc.). The extensive use of high doses of Ag NPs may cause serious adverse effects on both human health and the environment. Therefore, a clear understanding of the toxicities of these Ag NPs in human organs should be considered systematically by considering various doses and their adverse side effects associated with size, shape, and other morphological characteristics as the Ag NPs with less than 10 nm might have more potential to kill bacterial through penetration inside the bacteria.

More importantly, such outcomes will prelude in creating a great platform to combat the different infectious pathogen-based diseases. Moreover, more sensitive detection methods are required for avoiding the potential risks to human health. Without a doubt, it is also noteworthy that with the numerous applications of Ag and Ag NP-based antimicrobial agents that are being explored in various sectors, the impact of Ag NPs toxicity on human health and the environment should be considered as a significant potential risk factor.
